# Antiepileptic drugs in critically ill patients

**DOI:** 10.1186/s13054-018-2066-1

**Published:** 2018-06-07

**Authors:** Salia Farrokh, Pouya Tahsili-Fahadan, Eva K. Ritzl, John J. Lewin, Marek A. Mirski

**Affiliations:** 1 Department of Pharmacy, The Johns Hopkins Hospital, 600 N. Wolfe Street, Carnegie 180, Baltimore, MD 21287 USA; 2 Department of Neurology, The Johns Hopkins Hospital, Baltimore, MD USA; 3 Department of Anesthesiology and Critical Care Medicine, The Johns Hopkins Hospital, Baltimore, MD USA; 4 Department of Medicine, Virginia Commonwealth University School of Medicine, INOVA Campus, Falls Church, VA USA

**Keywords:** Seizure, Antiepileptic drugs, Critical care, Drug-drug Interaction, Pharmacokinetics, Pharmacodynamics

## Abstract

**Background:**

The incidence of seizures in intensive care units ranges from 3.3% to 34%. It is therefore often necessary to initiate or continue anticonvulsant drugs in this setting. When a new anticonvulsant is initiated, drug factors, such as onset of action and side effects, and patient factors, such as age, renal, and hepatic function, should be taken into account. It is important to note that the altered physiology of critically ill patients as well as pharmacological and nonpharmacological interventions such as renal replacement therapy, extracorporeal membrane oxygenation, and target temperature management may lead to therapeutic failure or toxicity. This may be even more challenging with the availability of newer antiepileptics where the evidence for their use in critically ill patients is limited.

**Main body:**

This article reviews the pharmacokinetics and pharmacodynamics of antiepileptics as well as application of these principles when dosing antiepileptics and monitoring serum levels in critically ill patients. The selection of the most appropriate anticonvulsant to treat seizure and status epileptics as well as the prophylactic use of these agents in this setting are also discussed. Drug-drug interactions and the effect of nonpharmacological interventions such as renal replacement therapy, plasma exchange, and extracorporeal membrane oxygenation on anticonvulsant removal are also included.

**Conclusion:**

Optimal management of antiepileptic drugs in the intensive care unit is challenging given altered physiology, polypharmacy, and nonpharmacological interventions, and requires a multidisciplinary approach where appropriate and timely assessment, diagnosis, treatment, and monitoring plans are in place.

## Background

The incidence of seizures in general intensive care units (ICUs) ranges from 3.3% to 34% [1]. Risk factors include common diagnoses such as brain tumor, head trauma, stroke, history of seizure, electrolyte abnormalities, hypoglycemia, infections, and drug overdose or withdrawal [2]. Thus, ICU management of antiepileptic drugs (AEDs) is routinely practiced by intensive care providers.

Selection of the most effective AED with the least adverse events, however, can often be challenging. About a third of patients with seizures fail on monotherapy, necessitating two or more AEDs [3]. Several factors commonly seen in the critical care setting, such as polypharmacy, unpredictable medication pharmacokinetics, and implementation of a variety of nonpharmacological interventions, may lead to drug-drug interactions, elevated risk for drug toxicity, and subtherapeutic drug serum levels; these are discussed in this review.

## Pharmacodynamics and pharmacokinetics of AEDs

### Pharmacodynamics

AEDs depress abnormal neuronal firing by various mechanisms of action including altering ion channel activity, enhancing gamma-aminobutyric acid (GABA)-mediated inhibition, or reducing glutamate-mediated excitation. While some AEDs have a single mechanism of action, others have multiple mechanisms of action and, in some, the exact mechanism of action is not yet known (Table 1).

**Table 1:** Mechanism of action of antiepileptic drugs [113]

Antiepileptic drug agent	Presumed mechanism of action
Brivaracetam	SV2A modulation
Carbamazepine	Na^+^ channel blockade
Clobazam	GABA potentiation
Clonazapam	GABA potentiation
Diazepam	GABA potentiation
Fosphenytoin/phenytoin	Na^+^ channel blockade
Lacosamide	Enhanced slow inactivation of voltage-gated Na^+^ channels
Lamotrigine	Na^+^ channel blockade
Levetiracetam	SV2A modulation
Lorazepam	GABA potentiation
Midazolam	GABA potentiation
Oxcarbazepine	Na^+^ channel blockade
Pentobarbital	GABA potentiation
Perampanel	AMPA glutamate receptor antagonist
Phenobarbital	GABA potentiation
Topiramate	Na^+^ channel blockade, GABA potentiation, AMPA/Kainate glutamate antagonist
Valproic acid	GABA potentiation, glutamate (NMDA) inhibition, sodium channel and T-type calcium channel blockade
Zonisamide	Na^+^ and Ca^2+^ channel blockade

*AMPA*, alpha-amino-3-hydroxy-5-methyl-4-isoxazolepropionic acid, *GABA* gamma-aminobutyric acid, *NMDA*, *N*-methyl-d-aspartate, *SV2A* synaptic vesicle glycoprotein 2A

When using multiple AEDs, it is reasonable to select medications with different mechanisms of action.

### Pharmacokinetics (absorption, distribution, metabolism, excretion)

Alterations in normal physiology and the physical properties of medications can affect the rate and extent of enteral absorption of medications in the ICU, leading to a need for parenteral administration. Deranged gastrointestinal absorption may be expected in circumstances of decreased blood flow, intestinal atrophy, dysmotility, and interactions with enteral nutrition. Pharmacokinetic parameters of selected AEDs, including their oral bioavailability, are summarized in Table 2.

**Table 2:** Bioavailability and pharmacokinetic data of antiepileptic drugs [113]

Antiepileptic drug agent	Bioavailability (PO formulation)	Protein binding	Half-life	Metabolism	Elimination
Brivaracetam	Almost completely absorbed	≤ 20%	~ 9 h	Hydrolysis (primary route) CYP2C19	≥ 95% renally
Carbamazepine	75–85% Total daily intravenous dose should be equivalent to 70% of previous total daily oral dose	75–95%	Range: 30–60 h After autoinduction: 12–17 h	> 90% by CYP3A4	72% renally
Clobazam	87%	80–90%	36 to 42 h	Hepatic via CYP3A4 and to a lesser extent via CYP2C19	82% renally
Clonazapam	~ 90%	~ 85%	17–60 h	Glucuronide and sulfate conjugation	< 2% renally as unchanged drug
Diazepam	> 90%	98%	Parent drug: ~ 60 to 72 h; metabolite ~ 152 to 174 h	CYP3A4 and 2C19	Renally
Eslicarbazepine	> 90%	< 40%	13–20 h	Hydrolytic first-pass metabolism	90% renally
Fosphenytoin	100% (intramuscular formulation)	95–99%	12–28.9 h	CYP2C9 CYP2C19	Renally
Lacosamide	100%	< 15%	13 h	CYP3A4, CYP2C9, CYP2C19	95% renally
Lamotrigine	98%	55%	25–70 h	Conjugation	94% renally
Levetiracetam	100%	< 10%	6–8 h	Hydrolysis	66% renally
Lorazepam	90%	~ 91%	12–18 h	Conjugation	88% renally
Midazolam					
Oxcarbazepine	Readily absorbed	40%	Active metabolite: 9–11 h	Glucoronidation	95% renally
Pentobarbital	–	45% to 70%	15–50 h	Hydroxylation and glucuronidation	< 1% renally as unchanged drug
Perampanel	Completely and rapidly absorbed	95%	~ 105 h	CYP 3A4/5 primary; lesser extent CYP 1A2/2B6	22% renally
Phenytoin	20–90%	90–95%	7–42 h	CYP2C9, 2C19 (major) and 3A4 (minor)	< 5% renally as unchanged drug
Phenobarbital	~ 95–100%	50%	Longest half-life 46–136 h	CYP450 and UGT mediated	25–50% renally
Topiramate	80%	15–41%	IR: 21 h ER: 31–56 h	No extensive metabolism	70% renally
Valproic acid	90%	80–90%	9–16 h	CYPs 2C9, 2C19, 2A6, UGT-glucuronidation	70–80% renally
Zonisamide	Rapid and complete absorption	40%	50–68 h	CYP 3A4	60% renally

*CYP* Cytochrome, *ER* Extended Release, *IR* Immediate Release, *PO* By mouth, *UGT* Uridine diphosphate-glucuronyltransferase

Changes in serum pH as well as both respiratory and renal failure affect the ionized state of many drugs, affecting their penetration across lipophilic-based membranes such as the blood-brain barrier. Bioavailability of active drugs is also affected by alterations in volume of distribution for hydrophilic medications, while hypoalbuminemia increases the unbound (free) fraction of highly albumin-bound medications.

Although drugs are commonly metabolized to less active compounds, prodrugs such as oxcarbazepine and fosphenytoin must be metabolized into their active forms carbamazepine and phenytoin, respectively. Drug metabolism generally occurs in two phases. Phase I involves nonsynthetic reactions to form a modified group. A cytochrome P450 (CYP450) enzyme is frequently involved in such oxidative reactions. Phase II involves synthetic reactions to conjugate the metabolite with an endogenous substance. Metabolism for most anticonvulsants occurs primarily in the liver and is dependent on hepatic blood flow, enzyme activity, and protein binding. Although critical illness often inhibits the CYP450 isoenzymes, drug metabolism may be enhanced over several days as in the cases of pentobarbital and phenytoin, resulting in potential subtherapeutic concentrations [4–6]. Induced hypothermia will also reduce the systemic clearance of many medications mediated by CYP450 (such as phenytoin or propofol), between 7 and 22% for every degree of Celsius below 37 [7].

Regardless of the route of administration, renal elimination of parent drugs or metabolites is the primary excretory pathway for most drugs. For patients on renal replacement therapy (RRT), the type of dialysis that is performed and the frequency/duration should also be considered (see section 8, special therapeutic considerations).

## Dosing and therapeutic drug monitoring in the ICU setting

Dosing of AEDs should be individualized to achieve seizure control with minimal adverse effects. The 1-h postloading dose is commonly recommended as a time to measure peak serum concentration. Pharmacokinetic alterations are frequently observed in critically ill patients; hence, frequent therapeutic drug monitoring (TDM) may be required. Basic dosing and TDM recommendations for AEDs commonly used in the ICU are summarized below and in Table 3.

**Table 3:** Dosing and monitoring of commonly used antiepileptic drugs [8, 11, 15, 16]

Antiepileptic drug	Dose	Therapeutic drug level to treat epilepsy (μg/mL)	Monitoring	TDM clinical pearls
Phenytoin	Load: 15–20 mg/kg Maintenance: 4–6 mg/kg/day	10–20	1 h postload or ~ 7–10 days after initiation of maintenance dose (may check earlier within 2–3 days in seizing patients to ensure their metabolism is not significantly different from average patient population)	At total concentrations > 20 μg/mL, nystagmus may occur. In concentrations > 30 μg/mL, ataxia, slurred speech, and incoordination can be observed. If total concentrations are above 40 μg/mL, coma is possible. At concentrations > 50–60 μg/m drug induced seizures may occur
Valproic acid	Load: 20–40 mg/kg Maintenance: 10–15 mg/kg/day	50–100 (levels as high as 175 are used in RSE)	1 h postload or 2–4 days after initiation of maintenance dose	At total concentrations > 75 μg/mL lethargy and ataxia may occur. In concentrations > 100 μg/mL tremor is observed. Coma may occur if total serum concentrations are above 175 μg/mL. Thrombocytopenia is a dose-related side effect that can be limited by reducing the dose
Phenobarbital	Load: 20 mg/kg Maintenance: 1.5–2 mg/kg/day (dose adjustment may be required in liver impairment due to reduced clearance)	15–40 (higher levels may be utilized in RSE)	1 h postload or 4–7 days after initiation of maintenance dose	CNS depression is a dose-related side effect. In concentrations > 60 μg/mL respiratory depression may occur
Pentobarbital	Load: 5–15 mg/kg Maintenance: 0.5–5 mg/kg/h	1–5 (rarely used to assess clinical efficacy or toxicity)	May be used after discontinuation to monitor the residual effects of the drug	Drug levels have not been correlated with electroencephalography CNS depression, respiratory depression, and hemodynamic instability are dose-related side effects

*CNS* central nervous system, *RSE* Refractory Status Epilepticus, *TDM* therapeutic drug monitoring

### Phenytoin/fosphenytoin

Phenytoin has an average half-life of 24 h, but this ranges from 7 to 40 h, increasing with dose escalation due to its nonlinear kinetics [8]. Phenytoin is insoluble in water and is dissolved in a basic solution including ethylene glycol, the mixture being linked to tissue necrosis (“purple hand syndrome”) if extravasated [9]. Fosphenytoin, the prodrug of phenytoin, is water soluble and hence free of the toxic emulsion. This difference in solubility allows intramuscular and faster intravenous administration of fosphenytoin, but the need for plasma conversion to the active drug (phenytoin) results in a comparable time to peak plasma levels when compared with phenytoin administration itself [10].

Because phenytoin follows nonlinear or saturable metabolism pharmacokinetics, it is possible to attain excessive concentrations much easier than medications that follow linear pharmacokinetics. Phenytoin TDM is therefore clinically important in the critically ill and should be followed closely.

At normal serum levels, patients may experience minor central nervous system depression and adverse effects such as nystagmus, drowsiness, or fatigue. Beyond the normal target range, ataxia, slurred speech, and incoordination often occurs. Drug-induced seizure activity has been reported at concentrations over 50–60 μg/mL [8].

Typically, protein binding accounts for 90% of total plasma concentrations, hence the therapeutic range for unbound phenytoin (free) concentrations is 1–2 μg/mL. In patients suspected of having altered drug plasma protein binding, monitoring of free phenytoin serum concentration is of value.

### Valproic acid

Although the accepted therapeutic range for total valproic acid concentration for seizure therapeutics is 50–100 μg/mL, levels up to 175 μg/mL have been suggested in cases of refractory status epilepticus (SE) cases. Concentration-related side effects include ataxia, lethargy, tremor, and coma [11]. The common adverse effects—thrombocytopenia and hyperammonemia (via carnitine depletion)—can often be limited by dose reduction and by carnitine replacement in the latter condition. Due to significant interpatient differences in valproic acid metabolism, there is a poor correlation between valproic acid dose and total serum concentrations [11, 12].

Valproic acid is highly (90–95%) protein bound and is saturable within the therapeutic range which results in higher unbound fractions at higher concentrations. Although not often monitored, a therapeutic free valproic acid range of 2.5–10 μg/mL can be used as an initial guide [11].

### Phenobarbital

Phenobarbital has a long half-life of approximately 100 h, which limits its use when short-term AED use is desired. As with other AEDs, treatment of SE in particular may require higher than normal dosing, and this may reach upwards of even 10 mg/kg/day (serum levels of > 100 μg/mL) with solid efficacy demonstrated [13]. Concentration-related adverse effects of phenobarbital are sedation, confusion, and lethargy, with high doses leading to obtundation and respiratory depression [14]. As phenobarbital is only about 50% protein-bound, free drug monitoring is not warranted. However, in severe hepatic impairment (Child-Pugh score > 8) a decrease of 25–50% in the initial daily maintenance dose may be required [15].

### Pentobarbital

Pentobarbital is often used in the ICU setting to treat SE or elevated intracranial pressure. Initiation of pentobarbital involves sequential bolus doses followed by a continuous infusion [16, 17]. The average half-life of pentobarbital in adults is reported to be about 22 h and it is 20–45% protein-bound. Serum pentobarbital TDM (reference range is 1–5 μg/mL) is of limited utility in determining treatment clinical response or toxicity; the direct measure of intracranial pressure (ICP) control or inducement of “burst suppression” by electroencephalography (EEG) are monitored instead. Serum concentrations, however, may be useful in assessing residual effects of pentobarbital-induced coma once discontinued [18].

## Drug-drug interactions

### AED-AED drug interactions

Administration of multiple AEDs is common in the ICU to manage refractory seizures and SE. Satisfactory seizure control is not achieved in 30–40% of patients with monotherapy, making it necessary to use two or more AEDs [19, 20]. Although synergistic anticonvulsant activity between medications may be desirable, concurrent use of multiple AEDs generally increases the risk of drug interactions. Table 4 lists common AED-AED drug-drug interactions.

**Table 4:** AED-AED drug interactions [113–116]

	CBZ	CNP	DZP	LAC	LAM	MDZ	OXZ	PEN	PMP	PHB	PHT	PFL	TOP
ZON	↓ZON			↓LAC			↓ZON	↓ZON		↓ZON	↓ZON		↓ZON
	↑CBZ												
VPA	↓VPA	? risk of absence seizures			↑ LAM		↓OXZ		↑VPA	↑PHB	↑PHT	↑PFL	↓VPA
	↑CBZ									↓VPA	↓VPA		
TOP	↓VPA		↑ DZP			↓MDZ		↓TOP	↓ PMP	↓TOP	↑PHT		
	↓TOP										↓TOP		
PHT	↓↑CBZ		↓ DZP	↓LAC	↓ LAM	↓MDZ		↓PEN	↓ PMP	↓PHT			
	↓↑PHT												
PHB	↓CBZ		↓ DZP		↓ LAM	↓MDZ	↓OXZ						
PEN	↓CBZ		↓ DZP			↓MDZ							
OXZ	↓CBZ		↑↓DZP			↓MDZ							
	↓OXZ												
MDZ	↓MDZ												
LAM	↓LAM												
LAC	↓LAC												
DZP	↓DZP												
	↑CBZ												
CNP													
BRV	↓BRV												

↑ and ↓ indicate increased and decreased levels and/or effects, respectively

*AED* antiepileptic drug, *CBZ* carbamazepine, *CNP* clonazepam, *GBT* gabapentine, *GTC* generalized tonic-clonic, *LAC* lacosamide, *LAM* lamotrigine, *LEV* levetiracetam, *OXZ* oxcarbazepine, *PMP* perampanel, *PHB* phenobarbital, *PHT* phenytoin, *PGB* pregabalin, *TOP* topiramate, *VPA* valproic acid, *ZON* zonisamide

### AED-nonAED interactions

Interactions of AEDs with other agents is seen more frequently with drugs that are metabolized in the liver, and therefore are more common with older AEDs. Tables 5 and 6 describe AED-nonAED and nonAED-AED interactions.

**Table 5:** Interaction of selected antiepileptic drugs with commonly used medications in the intensive care unit [113, 115, 117–124]

Antiepileptic drug	Therapeutic group	Selected examples
Phenytoin, phenobarbital, carbamazepine	Psychotropic agents	↓ Amitriptyline, nortriptyline, imipramine, bupropion, paroxetine, citalopram, Haloperidol, chlorpromazine, olanzapine, risperidone, quetiapine, ziprasidone
Valproic acid		↑ Amitriptyline, nortriptyline, paroxetine
Topiramate		↑ Haloperidol
Phenytoin, phenobarbital, carbamazepine	Antimicrobials	↓ Doxycycline, metronidazole, itraconazole, retrovirals
Valproic acid		↑ Zidovudine
Phenytoin	Cardiovascular agents	↓ Amiodarone, nimodipine, diltiazem, verapamil, ticagrelor, atorvastatin, dabigatran, apixaban, rivaroxaban (↑ warfarin effects with phenytoin load, ↓ warfarin effects with maintenance dose of phenytoin)^a^
Lacosamide		Diltiazem, verapamil (risk of atrioventricular block/bradycardia), ↓ warfarin
Carbamazepine		↓ Nimodipine, diltiazem, verapamil, ticagrelor, atorvastatin, warfarin, dabigatran, apixaban and rivaroxaban
Phenobarbital		↓ Nimodipine, atorvastatin
Valproic acid		↑ Nimodipine, warfarin
Phenytoin, phenobarbital, carbamazepine	Analgesics	↓ Fentanyl, methadone
Phenytoin, phenobarbital, carbamazepine	Immunosuppressant	↓ Cyclosporine, sirolimus, tacrolimus, corticosteroids

↑ and ↓ indicate increased and decreased levels and/or effects, respectively

^a^The interaction between phenytoin and warfarin is complicated and unpredictable. Close monitoring of the international normalized ratio (INR) as well as serum phenytoin levels are critical if this combination is clinically necessary. Upon initiation of phenytoin, there may be transient increases in the INR as a result of protein binding displacement of warfarin by phenytoin, and enhanced anticoagulant effect. This may be followed by a reduction in anticoagulant activity as result of phenytoin’s induction of warfarin metabolism [125, 126]

**Table 6:** Interaction of selected therapeutic classes with antiepileptic drugs [115, 117, 127–132]

Therapeutic class	Selected examples	Antiepileptic drugs
Psychotropic agents	Fluoxetine, sertraline, trazodone	↑ Phenytoin
	Trazodone, fluoxetine, risperidone	↑ Carbamazepine
	Sertraline	↑ Valproic acid
Antimicrobials	Erythromycin, clarithromycin, ketoconazole, fluconazole	↑ Carbamazepine
	Ritonavir	
	Sulfonamides	↑ Phenytoin
	Carbapenems (imipenem, doripenem, meropenem, ertapenem)	↓ Valproic acid
Cardiovascular agents	Amiodarone	↑ Phenytoin (a dose reduction of approximately 25% is recommended)
	Diltiazem	↑ Carbamazepine, phenytoin (risk of toxicity)
	Clopidogrel	↑ Phenytoin
Analgesics	Acetaminophen	↓ Lamotrigine
Immunosuppressants	Methotrexate	↓ Valproic acid, carbamazepine

↑ and ↓ indicate increased and decreased levels and/or effects, respectively

## Seizure prevention in the ICU

### Traumatic brain injury

The incidence of post-traumatic seizures accounts for 6% of overall symptomatic epilepsy [21–23] and is estimated to occur in up to 15% and 53% in civilian and military populations, respectively [24, 25]. Clinical studies have divided post-traumatic seizures into either immediate (< 24 h), early (within 1 week, with a seizure incidence of 2.1–16.9%), or late (with an incidence of 1.9% to > 30.0%) [22, 23, 26]).

Early seizures occur in about 10–15% of patients not treated with anticonvulsants, and this incidence can be reduced to about 2–3% with the use of phenytoin [27]. There is, however, little evidence that such use reduces the occurrence of late seizures or the incidence of death and neurologic disability [28]. It thus appears that anticonvulsants are suppressive during the early period following traumatic brain injury (TBI), but are not prophylactic for the time period beyond the first week. The American Academy of Neurology (AAN) [29] and the Brain Trauma Foundation [30] advocate prophylactic use of AED only during the first 7 days after a head injury [31, 32]. Phenytoin has been shown to decrease the incidence of early post-traumatic seizures [30] and to be more cost-effective than levetiracetam [33]. Although levetiracetam was shown in small early studies to be as effective as phenytoin [33, 34], there are recent data suggesting a lack of any prophylactic action in a comparative study of levetiracetam versus no prophylaxis [35]. A trial that incorporated a blinded design and the use of continuous electroencephalography (cEEG) monitoring demonstrated no difference in early or late seizure incidence between drugs, but did favor levetiracetam over phenytoin in the 6-month Glasgow Outcome score measure [36].

The use of valproic acid, as compared with phenytoin, failed to demonstrate benefit and its use correlated with higher mortality [37]. However, a recent Cochrane analysis failed to discriminate between phenytoin and other anticonvulsants with regards to reduced incidence or mortality [37].

### Ischemic stroke

Seizures following ischemic stroke occur within an incidence range of 4% to 14% [38–40]. According to a large recent registry from France, early seizures are not associated with higher mortality or unfavorable outcomes at 1 month or at 1 year [41]. Patients with late-onset seizures have a greater than twice the risk for subsequent stroke, although the causal relationship is unclear [42].

Given the rather low incidence of seizures following most ischemic stroke syndromes, neither the American Heart Association (AHA) nor the American Stroke Association guidelines recommend prophylactic use of anticonvulsants following ischemic stroke [43]. However, some patients may be at greater risk for seizures. An increase in seizure risk has been reported following cardioembolism [39], hyperglycemia [41], intravenous thrombolysis with alteplase [44], in patient with SE following poststroke seizures, and either large cortical or hemorrhagic infarction [38, 45–48].

If “prophylaxis” is considered, then the agent used should be selected for its adverse effect profile. In recent years there has been some interest in ascribing statins as a “first-line” anticonvulsant in stroke patients, although supportive data are by association only and this type of intervention is not readily practiced [49, 50].

### Intracerebral hemorrhage

In contrast to ischemic stroke, intracerebral hemorrhage (ICH) portends a somewhat higher risk of seizures, ranging from two- to seven-times that from ischemic stroke [38, 47, 51, 52]. If cEEG is performed during the acute admission period, nonconvulsive epileptiform activity is commonly observed and may affect more than 20% of ICH patients [53–56]. As expected, lobar or supratentorial ICHs have the highest incidence of seizures; those in the posterior fossa have almost zero [38, 54]. Surgical evacuation confers an increased incidence, and subarachnoid or subdural blood also increases the risk for seizures [57].

Prophylactic anticonvulsant treatment in ICH patients is currently not strongly advocated. cEEG is suggested by some for ICH patients with a depressed mental status out of proportion to that expected for the severity of the stroke [58]. Levetiracetam has become the most widely prescribed agent despite a lack of evidence [59], likely due to its ease of use. Uncontrolled study data have suggested that phenytoin confers worse outcomes than others (primarily against levetiracetam) [60–62], but these data are not supported by other recent studies [63, 64].

### Subarachnoid hemorrhage

Early and late-onset seizures are observed in patients with subarachnoid hemorrhage (SAH). Risk factors include the severity of SAH, concomitant intracerebral or intraventricular blood, and treatment with craniotomy [65]. A recent study noted a 10% incidence of seizures, with almost half (47%) occurring within the initial 24 h [66]. Interestingly, the use of prophylactic anticonvulsants did not reduce the overall risk. As seizures dramatically increase cerebral blood flow and blood pressure, rebleeding from a recently ruptured aneurysm during seizures may pose a serious risk.

Current guidelines from the AHA state that the use of anticonvulsants for a brief period is reasonable, without specific drug recommendation [67]. Beyond the immediate posthemorrhagic period, routine use of anticonvulsants is advocated by the AHA only in patients with a high risk for delayed seizures, such as prior seizure, intracerebral hematoma, intractable hypertension, infarction, or middle cerebral artery aneurysm. The Neurocritical Care Society (NCS) specifically recommended against the routine use of phenytoin for prophylaxis [68].

### Brain tumors

The incidence of seizures in cerebral malignancy broadly ranges from about 10% to as high as 90%, and is correlated with tumor type (glioma and oligodendroglioma being amongst the highest risk) and also the rate of progression, with slow growing tumors tending towards higher seizure incidence [69]. Prophylactic use of AEDs in brain tumors remains controversial. There are strikingly little data in support of seizure prophylaxis for brain tumor patients [70–72], and guidelines by the AAN and the European Federation of Neurosciences do not support this practice [73, 74]. Since these guidelines were written prior to the widespread use of cEEG and prior to the introduction of newer, less toxic anticonvulsants (levetiracetam, lamotrigine, lacosamide, and so forth), some have suggested that such a recommendation should be reassessed [75].

Whenever patients require adjuvant chemotherapy, it is prudent to avoid the use of potent CYP3A4 coenzyme inducers such as carbamazepine, phenytoin, and phenobarbital because of the risks of compromising concurrent chemotherapy [74]. Based on more recent evidence, more patients are being managed with levetiracetam than phenytoin or sodium valproate [71], but evidence of superior efficacy remains absent [72].

### Cerebral venous and sinus thrombosis

Seizures manifest as a common presentation, occurring in 29% to 50% of patients with cerebral venous sinus thrombosis [76, 77]. Approximately a third to one half of seizures occur early—as the presenting symptom or within 2 weeks of diagnosis, [77–79]. Despite the lack of controlled studies, prophylaxis appears prudent where forthcoming seizures appear highly probable. There is some evidence that early seizures are predictive of a remote epilepsy condition [80] but, in and of themselves, seizures in the context of cerebral venous sinus thrombosis do not appear to be associated with death or 6-month worse outcomes [81].

## Seizure control in the ICU

The widespread use of cEEG has made it apparent that seizures are more common in the ICU patients than previously thought, with estimates for seizures and SE ranging from 19% to 27% [82, 83]. Bauer and Trinka introduced the distinction between nonconvulsive status epilepticus (NCSE) proper and comatose NCSE [84]. This distinction is both important and useful as it acknowledges that treatment as well as prognosis are dependent on the underlying epileptic syndrome in the former, yet on the underlying cause of the coma in the latter. In the ICU patient population, the electrographic seizure patterns can be less distinct and harder to identify. Thus, a body of literature on the nature of, and the best treatment approach for, the “ictal-interictal continuum” has emerged and now dominates the literature. For recent reviews, see Sivaraju and Gilmore 2016 [85] and Rodríguez et al. 2016 [86].

As a general principle, the current consensus is that treatment of even a single ICU seizure should occur in order to prevent escalation into SE. Once airway, breathing, circulation, and “dextrose” (the “ABCDs”) have been addressed, pharmacologic seizure treatment should occur immediately. Several authors suggest an algorithm that escalates from first-line to second-line treatment in the time span of 30 min [87, 88]. Once urgent first- and second-line agents are prescribed during the first 30 min, persistent SE should be considered refractory, and this should prompt the use of intravenous anesthetic therapy. SE is a neurological emergency and swift intervention is of the essence. While convulsive and nonconvulsive SE should be initially approached in the same way, once the need for intubation arises it is generally accepted that NCSE may warrant a less aggressive and more individualized approach to treatment with anesthetic agents (third-line agents) compared with convulsive SE.

Available evidence uniformly suggests that benzodiazepines remain the drugs of choice for the immediate control of seizures of any kind. The efficacy of intravenous lorazepam was demonstrated by Treiman et al. in 1998 [89]. Lorazepam was most effective in the initial treatment of convulsive SE when compared with phenobarbital and diazepam plus phenytoin, although the latter were also effective. Other routes for the administration of benzodiazepines have successfully been explored, particularly for the therapy of seizures encountered outside the ICU. Silbergleit et al. reported that intramuscular midazolam was at least as safe and effective as intravenous lorazepam for prehospital seizure cessation [90], while McIntyre et al. demonstrated the efficacy of buccal midazolam [91].

Although an open-label use study purported to suggest equipoise between lorazepam and levetiracetam in the treatment of SE [92], a randomized, double-blind, placebo-controlled trial (SAMU Keppra Trial) failed to show benefit from adding intravenous levetiracetam to clonazepam in the treatment of generalized convulsive SE [93].

To monitor for ongoing seizure activity after convulsions may have ceased, cEEG is crucial. Second-line therapy for SE should be initiated within 30 min if first-line treatment has failed. Agents used as second-line therapy include fosphenytoin, valproic acid, phenobarbital, and levetiracetam. Evidence for the superiority of one over the other is generally lacking [94]. Nonetheless, phenytoin has been the most studied drug in SE and is therefore often listed as the preferred choice over the others. Valproic acid is notable for being the only agent that has shown a trend towards superior efficacy in some studies but this has not been confirmed in a large-scale trial [95]. Levetiracetam has a favorable side-effect profile and is therefore increasingly prescribed as a second-line drug. Phenobarbital is often avoided due to its long half-life, although data support its efficacy [89]. Lacosamide has recently shown promise in small case studies [96], while brivaracetam is currently under investigation [97].

If second-line agents are ineffective, treatment is typically escalated to anesthetic agents. For patients in NCSE, however, the decision whether or not to intubate and sedate the patient will usually be made on a case-by-case basis. There is some evidence to suggest that uncontrolled NCSE will result in brain damage over time [98]. Furthermore, DeLorenzo et al. noted that persistent NCSE after convulsive status may carry a worse prognosis than other forms of nonconvulsive status and perhaps should be treated more aggressively, although the presence of NCSE may simply be a marker for more severe brain injury [99]. Agents used for continuous infusion are midazolam, propofol, and pentobarbital, with no data suggesting the superiority of one over the other [100]. Ultimately, it is important not only to treat the patient with an anesthetic agent but to also continue maintenance therapy with appropriate AEDs to allow transition to a well-tolerated, long-term antiepileptic regimen.

## Drugs lowering seizure threshold

Polypharmacy is common in the ICU and can contribute to adverse drug effects such as seizures by lowering the seizure threshold or via drug-drug interactions that may reduce the blood concentration of an AED the patient may already be receiving (see the next section). Antibiotics, psychotropic agents, and analgesics have been all associated with seizures [101, 102] (Table 7).

**Table 7:** Selected list of medications associated with lower seizure threshold [113]

Antibiotics	Psychotropic agents	Analgesics	Neurostimulants	Miscellaneous agents
Cefepime	Bupropion	Meperidine^b^	Amantadine	Baclofen
Erythromycin	Haloperidol	Tramadol	Amphetamines	Flumazenil
Imipenem^a^	Phenothiazines		Bromocriptine	
Isoniazid	SSRIs			
Levofloxacin	TCAs			
Linezolid				
Meropenem				
Metronidazole				
Penicillins				

*SSRI* selective serotonin reuptake inhibitor, *TCA* tricyclic antidepressant

^a^Carbapenems have been reported to have the highest rate of seizure among all drugs

^b^Normeperidine, the active metabolite of meperidine, has been associated with seizures and accumulates in renal failure

## Special therapeutic considerations

### Extracorporeal removal of antiepileptic drugs

#### Antiepileptic drug removal by renal replacement therapies

The renal route of elimination, low protein binding, low volume of distribution, and molecular weight are physiochemical factors that impact the propensity for drug removal via dialysis (Table 8). Unfortunately, kinetic data on optimal dosing of AEDs in RRT are not readily available. Clearly such patients benefit from careful TDM and a multidisciplinary approach. There are extensive expert reviews available in the medical literature [103, 104].

**Table 8:** Factors impacting drug removal by renal replacement therapies

Drug-related factors	RRT-related factors
• Low protein binding	• Mode of dialysis (CRRT vs. IHD vs. others)
• Low volume of distribution	• RRT filter membrane (sieving coefficient)
• Predominantly renally eliminated	• Dialysis prescription (mode and flow rates)
• Low molecular weight	• Duration/frequency of renal replacement

*CRRT* continuous renal replacement therapy, *IHD* intermittent hemodialysis, *RRT* renal replacement therapy

##### Intermittent hemodialysis

The removal of most AEDs by intermittent hemodialysis (IHD) is usually well characterized in the drug’s labeled prescribing information or other references. Two common drugs that are removed by IHD and for which data recommendations exist are levetiracetam and lacosamide. In addition, while phenobarbital is predominantly hepatically metabolized, some reports suggest partial elimination by IHD prompting several experts to suggest supplemental dosing after IHD and monitoring of serum drug levels [103, 105, 106].

##### Continuous renal replacement therapy

There is insufficient evidence to recommend clear dosing guidelines for AEDs in continuous renal replacement therapy (CRRT), and more research is needed to appropriately guide therapy. AEDs that are eliminated via IHD will also be eliminated by CRRT, often times even more so (depending on the duration of CRRT as well as the mode and flow rates) thereby requiring higher doses. For drugs where serum levels are available, TDM is indicated. Estimations of drug removal can be performed to estimate the removal of medications by CRRT based on flow rates, as well as drug and filter properties. The reader is referred to other resources for a more comprehensive discussion on these methods [107, 108]. A recent review of the literature proposed dosing recommendations based on AED protein binding and the extent of renal versus hepatic elimination [104]. This is a reasonable approach, although clinicians should also factor in CRRT flow rates, the clinical condition of the patient, and other factors into their clinical decision making accordingly.

#### Antiepileptic drug removal by plasmapheresis

In the neurocritical care unit, plasmapheresis (PLEX) is often employed to manage neuroautoimmune diseases. Unlike RRT, which removes the free fraction of a drug, PLEX removes whole plasma which includes both the free fraction of a drug as well as the protein-bound portion. Thus, a key factor in determining the whole body removal of a drug with PLEX is its volume of distribution. Other factors can include the duration/frequency of PLEX and exchange volume, as well as the rate of intercompartmental equilibration. In general, drugs with low volumes of distribution (< 0.2 L/kg) predominantly reside in the vascular compartment and PLEX would be expected to remove a significant portion of the total body stores of these drugs. As for AEDs with high volumes of distribution such as phenytoin, PLEX appears to remove only about 2.5–10% of total body phenytoin [109, 110].

#### Antiepileptic drug removal by extracorporeal membrane oxygenation

Extracorporeal membrane oxygenation (ECMO) can impact the pharmacokinetics of drugs in a number of ways. ECMO circuits are well known to sequester drugs given the large surface area of the tubing and membranes which can result in an increase in the volume of distribution initially [111], while later releasing the drug into the circulation resulting in an unpredictable effect [112]. Lipophilic and highly protein-bound drugs are considered to be particularly vulnerable, but other physiochemical factors such as molecular weight, protein binding, and ionization may all play a role [113]. In addition, ECMO is typically associated with reduced drug clearance as a result of alterations of end-organ perfusion. ECMO is also often combined with some form of RRT, further complicating predictability. One case report suggests ECMO has little impact on the disposition of levetiracetam (which has a low volume of distribution and low protein binding) [114]. Where available, clinicians should monitor serum levels to guide dosing in patients on AEDs.

## Conclusion

Selection of the most appropriate AED in the ICU setting can be challenging for a variety of reasons. Older AEDs such as phenytoin, valproic acid, and phenobarbital are often used by clinicians due to their familiarity with these agents, intravenous formulations, and availability of evidence in certain clinical scenarios. Despite this, adverse effects of these agents, drug-drug interactions, and the need for TDM may limit their use. Newer agents such as levetiracetam and lacosamide are gaining popularity due to their relatively safe AED profile, fewer drug-drug interactions, and lack of need for TDM. The efficacy of these agents for seizure prophylaxis and as second-line treatment for SE, however, should be further evaluated in large randomized clinical trials.

## Abbreviations

AAN: American Academy of Neurology
AED: Antiepileptic drug
AHA: American Heart Association
cEEG: Continuous electroencephalography
CRRT: Continuous renal replacement therapy
CYP450: Cytochrome P450
ECMO: Extracorporeal membrane oxygenation
EEG: Electroencephalography
GABA: Gamma-aminobutyric acid
ICH: Intracerebral hemorrhage
ICP: Intracranial pressure
ICU: Intensive care unit
IHD: Intermittent hemodialysis
NCSE: Nonconvulsive status epilepticus
PLEX: Plasmapheresis
RRT: Renal replacement therapy
SAH: Subarachnoid hemorrhage
SE: Status epilepticus
TBI: Traumatic brain injury
TDM: Therapeutic drug monitoring

## References

[1] Varelas PN, Spanaki MV, Mirski MA (2013). Seizures and the neurosurgical intensive care unit. Neurosurg Clin N Am.

[2] Voils SA, Human T, Brophy GM (2014). Adverse neurologic effects of medications commonly used in the intensive care unit. Crit Care Clin.

[3] Lee JW, Dworetzky B (2010). Rational polytherapy with antiepileptic drugs. Pharmaceuticals.

[4] Boucher BA, Hanes SD (1998). Pharmacokinetic alterations after severe head injury. Clin Pharmacokinet.

[5] Boucher BA (1988). Phenytoin pharmacokinetics in critically ill trauma patients. Clin Pharmacol Ther.

[6] McKindley DS (1997). Effect of acute phase response on phenytoin metabolism in neurotrauma patients. J Clin Pharmacol.

[7] Arpino PA, Greer DM (2008). Practical pharmacologic aspects of therapeutic hypothermia after cardiac arrest. Pharmacotherapy.

[8] Bauer LA (2015). Phenytoin/fosphenytoin. Applied clinical pharmacokinetics, 3e.

[9] Spengler RF (1988). Severe soft-tissue injury following intravenous infusion of phenytoin: Patient and drug administration risk factors. Arch Intern Med.

[10] Millares-Sipin CA, Alafris A, Cohen H, Cohen H (2015). Phenytoin and fosphenytoin, in casebook. Clinical pharmacokinetics and drug dosing.

[11] Bauer LA (2015). Valproic acid. Applied clinical pharmacokinetics, 3e.

[12] Bauer LA (1985). Valproic acid clearance: unbound fraction and diurnal variation in young and elderly adults. Clin Pharmacol Ther.

[13] Mirski MA, Williams MA, Hanley DF (1995). Prolonged pentobarbital and phenobarbital coma for refractory generalized status epilepticus. Crit Care Med.

[14] Rogers SJ, Cavazos JE, DiPiro JT (2014). Chapter 40. Epilepsy. Pharmacotherapy: a pathophysiologic approach, 9e.

[15] Bauer LA (2015). Phenobarbital/primidone. Applied clinical pharmacokinetics, 3e.

[16] Eisenberg HM (1988). High-dose barbiturate control of elevated intracranial pressure in patients with severe head injury. J Neurosurg.

[17] Wermeling DP (1987). Pentobarbital pharmacokinetics in patients with severe head injury. Drug Intell Clin Pharm.

[18] Huynh F, Mabasa VH, Ensom MH (2009). A critical review: does thiopental continuous infusion warrant therapeutic drug monitoring in the critical care population?. Ther Drug Monit.

[19] Kwan P, Brodie MJ (2006). Combination therapy in epilepsy. Drugs.

[20] Kwan P, Brodie MJ (2001). Effectiveness of first antiepileptic drug. Epilepsia.

[21] Temkin NR (2009). Preventing and treating posttraumatic seizures: the human experience. Epilepsia.

[22] Herman ST (2002). Epilepsy after brain insult targeting epileptogenesis. Neurology.

[23] Pitkänen A, Bolkvadze T (2010). Head trauma and epilepsy. Epilepsia.

[24] Traumatic Brain Injury. Available from: http://www.cdc.gov/TraumaticBrainInjury/index.html. Accessed 14 Jan 2018.

[25] Lee S-T (1995). Early seizures after moderate closed head injury. Acta Neurochir.

[26] Frey LC (2003). Epidemiology of posttraumatic epilepsy: a critical review. Epilepsia.

[27] Temkin NR (1990). A randomized, double-blind study of phenytoin for the prevention of post-traumatic seizures. N Engl J Med.

[28] Schierhout G, Roberts I (1998). Prophylactic antiepileptic agents after head injury: a systematic review. J Neurol Neurosurg Psychiatry.

[29] Chang BS, Lowenstein DH (2003). Antiepileptic drug prophylaxis in severe traumatic brain injury. Neurology.

[30] Bratton S (2006). Guidelines for the management of severe traumatic brain injury. XIII Antiseizure prophylaxis. J Neurotrauma.

[31] Sundararajan K (2015). Anti-seizure prophylaxis in critically ill patients with traumatic brain injury in an intensive care unit. Anaesth Intensive Care.

[32] Cranley MR, Craner M, McGilloway E (2015). Antiepileptic prophylaxis following severe traumatic brain injury within a military cohort. J R Army Med Corps..

[33] Cotton BA (2011). Cost-utility analysis of levetiracetam and phenytoin for posttraumatic seizure prophylaxis. J Trauma Acute Care Surg.

[34] Jones KE (2008). Levetiracetam versus phenytoin for seizure prophylaxis in severe traumatic brain injury. Neurosurg Focus.

[35] Zangbar B (2016). Levetiracetam prophylaxis for post-traumatic brain injury seizures is ineffective: a propensity score analysis. World J Surg.

[36] Szaflarski JP (2010). Prospective, randomized, single-blinded comparative trial of intravenous levetiracetam versus phenytoin for seizure prophylaxis. Neurocrit Care.

[37] Temkin NR (1999). Valproate therapy for prevention of posttraumatic seizures: a randomized trial. J Neurosurg.

[38] Bladin CF (2000). Seizures after stroke: a prospective multicenter study. Arch Neurol.

[39] Szaflarski JP (2008). Incidence of seizures in the acute phase of stroke: a population-based study. Epilepsia.

[40] Bryndziar T (2016). Seizures following ischemic stroke: frequency of occurrence and impact on outcome in a long-term population-based study. J Stroke Cerebrovasc Dis.

[41] Hamidou B (2013). Prognostic value of early epileptic seizures on mortality and functional disability in acute stroke: the Dijon Stroke Registry (1985–2010). J Neurol.

[42] Cleary P, Shorvon S, Tallis R (2004). Late-onset seizures as a predictor of subsequent stroke. Lancet.

[43] Jauch EC (2013). Guidelines for the early management of patients with acute ischemic stroke. Stroke.

[44] Alvarez V (2013). Acute seizures in acute ischemic stroke: does thrombolysis have a role to play?. J Neurol.

[45] Reith J (1997). Seizures in acute stroke: predictors and prognostic significance. The Copenhagen Stroke Study.

[46] Lamy C (2003). Early and late seizures after cryptogenic ischemic stroke in young adults. Neurology.

[47] Beghi E (2011). Incidence and predictors of acute symptomatic seizures after stroke. Neurology.

[48] Rumbach L (2000). Status epilepticus in stroke: report on a hospital-based stroke cohort. Neurology.

[49] Guo J (2015). Statin treatment reduces the risk of poststroke seizures. Neurology.

[50] Siniscalchi A, Mintzer S (2015). Statins for poststroke seizures: the first antiepileptogenic agent?. Neurology..

[51] Lahti A-M (2017). Poststroke epilepsy in long-term survivors of primary intracerebral hemorrhage. Neurology.

[52] Giroud M (1994). Early seizures after acute stroke: a study of 1,640 cases. Epilepsia.

[53] Vespa PM (2003). Acute seizures after intracerebral hemorrhage: a factor in progressive midline shift and outcome. Neurology.

[54] Faught E (1989). Seizures after primary intracerebral hemorrhage. Neurology.

[55] Tatu L (2000). Primary intracerebral hemorrhages in the Besancon stroke registry. Eur Neurol.

[56] Claassen J (2007). Electrographic seizures and periodic discharges after intracerebral hemorrhage. Neurology.

[57] Garrett MC (2009). Predictors of seizure onset after intracerebral hemorrhage and the role of long-term antiepileptic therapy. J Crit Care.

[58] Morgenstern LB (2010). Guidelines for the management of spontaneous intracerebral hemorrhage. A guideline for healthcare professionals from the American Heart Association/American Stroke Association. Stroke.

[59] Naidech AM (2017). Evolving use of seizure medications after intracerebral hemorrhage: a multicenter study. Neurology.

[60] Naidech AM (2009). Anticonvulsant use and outcomes after intracerebral hemorrhage. Stroke.

[61] Messé SR (2009). Prophylactic antiepileptic drug use is associated with poor outcome following ICH. Neurocrit Care.

[62] Gilmore EJ (2016). Review of the utility of prophylactic anticonvulsant use in critically ill patients with intracerebral hemorrhage. Stroke.

[63] Sheth KN (2015). Prophylactic antiepileptic drug use and outcome in the ethnic/racial variations of intracerebral hemorrhage study. Stroke.

[64] Zandieh A (2016). Prophylactic use of antiepileptic drugs in patients with spontaneous intracerebral hemorrhage. J Stroke Cerebrovasc Dis.

[65] Maciel CB, Gilmore EJ (2016). Seizures and epileptiform patterns in SAH and their relation to outcomes. J Clin Neurophysiol.

[66] Panczykowski D (2016). Prophylactic antiepileptics and seizure incidence following subarachnoid hemorrhage. Stroke.

[67] Connolly ES (2012). Guidelines for the management of aneurysmal subarachnoid hemorrhage. Stroke..

[68] Diringer MN (2011). Critical care management of patients following aneurysmal subarachnoid hemorrhage: recommendations from the Neurocritical Care Society’s Multidisciplinary Consensus Conference. Neurocrit Care.

[69] Bauer R (2014). Treatment of epileptic seizures in brain tumors: a critical review. Neurosurg Rev.

[70] Garbossa D (2013). A retrospective two-center study of antiepileptic prophylaxis in patients with surgically treated high-grade gliomas. Neurol India.

[71] Kruer RM (2013). Changing trends in the use of seizure prophylaxis after traumatic brain injury: a shift from phenytoin to levetiracetam. J Crit Care.

[72] Zafar SN (2012). Phenytoin versus leviteracetam for seizure prophylaxis after brain injury—a meta analysis. BMC Neurol.

[73] Glantz M (2000). Practice parameter: anticonvulsant prophylaxis in patients with newly diagnosed brain tumors. Report of the Quality Standards Subcommittee of the American Academy of Neurology. Neurology.

[74] Soffietti R (2010). Guidelines on management of low-grade gliomas: report of an EFNS–EANO Task Force. Eur J Neurol.

[75] Wychowski T (2013). Considerations in prophylaxis for tumor-associated epilepsy: prevention of status epilepticus and tolerability of newer generation AEDs. Clin Neurol Neurosurg.

[76] Masuhr F (2006). Risk and predictors of early epileptic seizures in acute cerebral venous and sinus thrombosis. Eur J Neurol.

[77] Ferro J (2003). Seizures in cerebral vein and dural sinus thrombosis. Cerebrovasc Dis.

[78] Ferro JM (2008). Early seizures in cerebral vein and dural sinus thrombosis. Stroke.

[79] Mahale R (2016). Acute seizures in cerebral venous sinus thrombosis: what predicts it?. Epilepsy Res.

[80] Davoudi V, Saadatnia M (2014). Risk factors for remote seizure development in patients with cerebral vein and dural sinus thrombosis. Seizure.

[81] Kalita J, Chandra S, Misra UK (2012). Significance of seizure in cerebral venous sinus thrombosis. Seizure.

[82] Claassen J (2004). Detection of electrographic seizures with continuous EEG monitoring in critically ill patients. Neurology.

[83] Westover MB (2015). The probability of seizures during EEG monitoring in critically ill adults. Clin Neurophysiol.

[84] Bauer G, Trinka E (2010). Nonconvulsive status epilepticus and coma. Epilepsia.

[85] Sivaraju A, Gilmore EJ (2016). Understanding and managing the ictal-interictal continuum in neurocritical care. Curr Treat Options Neurol.

[86] Rodríguez V, Rodden MF, LaRoche SM (2016). Ictal–interictal continuum: a proposed treatment algorithm. Clin Neurophysiol.

[87] Costello DJ, Cole AJ (2007). Treatment of acute seizures and status epilepticus. J Intensive Care Med.

[88] Billington M, Kandalaft OR, Aisiku IP (2016). Adult status epilepticus: a review of the prehospital and emergency department management. J Clin Med.

[89] Treiman DM (1998). A comparison of four treatments for generalized convulsive status epilepticus. N Engl J Med.

[90] Silbergleit R (2012). Intramuscular versus intravenous therapy for prehospital status epilepticus. N Engl J Med.

[91] McIntyre J (2005). Safety and efficacy of buccal midazolam versus rectal diazepam for emergency treatment of seizures in children: a randomised controlled trial. Lancet.

[92] Misra U, Kalita J, Maurya P (2012). Levetiracetam versus lorazepam in status epilepticus: a randomized, open labeled pilot study. J Neurol.

[93] Navarro V (2016). Prehospital treatment with levetiracetam plus clonazepam or placebo plus clonazepam in status epilepticus (SAMUKeppra): a randomised, double-blind, phase 3 trial. Lancet Neurol.

[94] Brophy G (2012). Neurocritical Care Society Status Epilepticus Guideline Neurocrit Care Writing Committee. Guidelines for the evaluation and management of status epilepticus. Neurocrit Care.

[95] Misra U, Kalita J, Patel R (2006). Sodium valproate vs phenytoin in status epilepticus: a pilot study. Neurology.

[96] Höfler J, Trinka E (2013). Lacosamide as a new treatment option in status epilepticus. Epilepsia.

[97] Strzelczyk A (2017). Treatment of refractory and super-refractory status epilepticus with brivaracetam: A cohort study from two German university hospitals. Epilepsy Behav.

[98] Hirsch LJ, Gaspard N. Status epilepticus CONTINUUM: lifelong learning in neurology. Continuum. 2013;19(3, Epilepsy):767–94. 10.1212/01.CON.0000431395.16229.5a PMC10564021 23739110

[99] DeLorenzo R (1998). Persistent nonconvulsive status epilepticus after the control of convulsive status epilepticus. Epilepsia.

[100] Rossetti AO, Lowenstein DH (2011). Management of refractory status epilepticus in adults: still more questions than answers. Lancet Neurol.

[101] Miller AD (2011). Epileptogenic potential of carbapenem agents: mechanism of action, seizure rates, and clinical considerations. Pharmacotherapy.

[102] Pisani F (2002). Effects of psychotropic drugs on seizure threshold. Drug Saf.

[103] Asconape JJ (2014). Use of antiepileptic drugs in hepatic and renal disease. In: Miller J, Ferro JM, editors. Handbook of Clinical Neurology..

[104] Mahmoud SH (2017). Antiepileptic drug removal by continuous renal replacement therapy: a review of the literature. Clin Drug Investig.

[105] Porto I, John EG, Heilliczer J (1997). Removal of phenobarbital during continuous cycling peritoneal dialysis in a child. Pharmacotherapy.

[106] Palmer BF (2000). Effectiveness of hemodialysis in the extracorporeal therapy of phenobarbital overdose. Am J Kidney Dis.

[107] Schetz M (1995). Pharmacokinetics of continuous renal replacement therapy. Intensive Care Med.

[108] Reetze-Bonorden P, Bohler J, Keller E (1993). Drug dosage in patients during continuous renal replacement therapy. Pharmacokinetic and therapeutic considerations. Clin Pharmacokinet.

[109] Silberstein LE, Shaw LM (1986). Effect of plasma exchange on phenytoin plasma concentration. Ther Drug Monit.

[110] Ibrahim RB (2007). Drug removal by plasmapheresis: an evidence-based review. Pharmacotherapy.

[111] Elliott ES, Buck ML (1999). Phenobarbital dosing and pharmacokinetics in a neonate receiving extracorporeal membrane oxygenation. Ann Pharmacother.

[112] Shekar K (2012). Pharmacokinetic changes in patients receiving extracorporeal membrane oxygenation. J Crit Care.

[113] Shekar K (2015). Protein-bound drugs are prone to sequestration in the extracorporeal membrane oxygenation circuit: results from an ex vivo study. Crit Care.

[114] Nei SD (2015). Levetiracetam pharmacokinetics in a patient receiving continuous venovenous hemofiltration and venoarterial extracorporeal membrane oxygenation. Pharmacotherapy.

[115] Patsalos PN. Drug interactions with the newer antiepileptic drugs (AEDs)—part 2: pharmacokinetic and pharmacodynamic interactions between AEDs and drugs used to treat non-epilepsy disorders. Clinical pharmacokinetics. 2013;52(12):1045–1061. 10.1007/s40262-013-0088-z 23794036

[116] Patsalos PN, Perucca E. Clinically important drug interactions in epilepsy: general features and interactions between antiepileptic drugs. The Lancet Neurology. 2003;2(6):347–356. 10.1016/s1474-4422(03)00409-5 12849151

[117] Sancho J, Serratosa J. Antiepileptic drug interactions. The neurologist. 2008;14(6):S55–S65. 10.1097/01.nrl.0000340792.61037.40 19225371

[118] Schmidt D, Schachter SC. Drug treatment of epilepsy in adults. Bmj. 2014;348:g254. 10.1136/bmj.g254 24583319

[119] Perucca E. Clinically relevant drug interactions with antiepileptic drugs. British journal of clinical pharmacology. 2006;61(3):246–255. 10.1111/j.1365-2125.2005.02529.x PMC1885026 16487217

[120] French J, et al. Efficacy and tolerability of the new antiepileptic drugs I: Treatment of new onset epilepsy Report of the Therapeutics and Technology Assessment Subcommittee and Quality Standards Subcommittee of the American Academy of Neurology and the American Epilepsy Society. Neurology. 2004;62(8):1252–1260. 10.1212/01.wnl.0000123693.82339.fc 15111659

[121] Food U and D Administration. VIMPAT®(lacosamide) Prescribing Information. Accessed July, 2017.

[122] Ingelheim B. Pradaxa (dabigatran) package insert. Ridgefield, CT. 2012.

[123] Healthcare B. Xarelto (rivaroxaban) package insert. 2012.

[124] Squibb B.-M. Eliquis (apixaban) package insert. 2015.

[125] Nappi JM. Warfarin and phenytoin interaction. Ann Intern Med. 1979;90(5):852. 10.7326/0003-4819-90-5-852_1 434697

[126] Levine M, Sheppard I. Biphasic interaction of phenytoin with warfarin. Clin Pharm. 1984;3(2):200–3. 6723231

[127] Food U and D. Administration, Cordarone (Amiodarone HCL) Tablets: Package Insert. US Food and Drug Administration, Maryland. 2010.

[128] Macphee GA, et al. Verapamil potentiates carbamazepine neurotoxicity: a clinically important inhibitory interaction. The Lancet. 1986;327(8483):700–703. 10.1016/s0140-6736(86)91099-8 2870222

[129] Maoz E, et al. Carbamazepine neurotoxic reaction after administration of diltiazem. Archives of internal medicine. 1992;152(12):2503–2504. 1456863

[130] Bahls FH, Ozuna J, Ritchie DE. Interactions between calcium channel blockers and the anticonvulsants carbamazepine and phenytoin. Neurology. 1991;41(5):740–742. 10.1212/wnl.41.5.740 2027492

[131] I Johannessen, S. and C. Johannessen Landmark, Antiepileptic drug interactions-principles and clinical implications. Current neuropharmacology. 2010;8(3):254–267. 10.2174/157015910792246254 PMC3001218 21358975

[132] Park MK, et al. Reduced valproic acid serum concentrations due to drug interactions with carbapenem antibiotics: overview of 6 cases. Therapeutic drug monitoring. 2012;34(5):599–603. 10.1097/FTD.0b013e318260f7b3 22929406

